# PAM50 assay and the three-gene model for identifying the major and clinically relevant molecular subtypes of breast cancer

**DOI:** 10.1007/s10549-012-2143-0

**Published:** 2012-07-03

**Authors:** A. Prat, J. S. Parker, C. Fan, C. M. Perou

**Affiliations:** 1 Departament de Medicina, Universitat Autònoma de Barcelona, Barcelona, Spain; 2 Department of Genetics, University of North Carolina, Chapel Hill, NC USA; 3 Lineberger Comprehensive Cancer Center, University of North Carolina, CB# 7295, Chapel Hill, NC 27599 USA; 4 Department of Pathology & Laboratory Medicine, University of North Carolina, Chapel Hill, NC USA

**Keywords:** Breast cancer, Microarrays, PAM50, Prognosis, Gene expression

## Abstract

It has recently been proposed that a three-gene model (SCMGENE) that measures ESR1, ERBB2, and AURKA identifies the major breast cancer intrinsic subtypes and provides robust discrimination for clinical use in a manner very similar to a 50-gene subtype predictor (PAM50). However, the clinical relevance of both predictors was not fully explored, which is needed given that a ~30 % discordance rate between these two predictors was observed. Using the same datasets and subtype calls provided by Haibe-Kains and colleagues, we compared the SCMGENE assignments and the research-based PAM50 assignments in terms of their ability to (1) predict patient outcome, (2) predict pathological complete response (pCR) after anthracycline/taxane-based chemotherapy, and (3) capture the main biological diversity displayed by all genes from a microarray. In terms of survival predictions, both assays provided independent prognostic information from each other and beyond the data provided by standard clinical–pathological variables; however, the amount of prognostic information was found to be significantly greater with the PAM50 assay than the SCMGENE assay. In terms of chemotherapy response, the PAM50 assay was the only assay to provide independent predictive information of pCR in multivariate models. Finally, compared to the SCMGENE predictor, the PAM50 assay explained a significantly greater amount of gene expression diversity as captured by the two main principal components of the breast cancer microarray data. Our results show that classification of the major and clinically relevant molecular subtypes of breast cancer are best captured using larger gene panels.

**Electronic supplementary material:**

The online version of this article (doi:10.1007/s10549-012-2143-0) contains supplementary material, which is available to authorized users.

## Introduction

Over the years, global gene expression analyses have identified at least four intrinsic subtypes of breast cancer (Luminal A, Luminal B, HER2-enriched, and Basal-like) and a normal-like group with significant differences in terms of their risk factors, incidence, baseline prognoses and responses to systemic therapies [1–4]. In 2009, we reported a clinically applicable gene expression-based predictor that robustly identifies these main intrinsic subtypes by quantitative measurement of 50 genes (i.e., PAM50) [1]. Identification of these molecular subtypes using pathology-based surrogate definitions based upon hormone receptors (HRs), HER2 and Ki-67 expressions has been adopted by the 2011 St. Gallen Consensus Conference for treatment decision-making in early breast cancer [5], however, controversy exists as to whether these complex molecular subtypes can be effectively captured using four or less biomarkers.

Recently, Haibe-Kains et al. [6] reported a mRNA expression predictor that classifies tumors into four molecular entities (ER+/HER2−/Low Proliferative, ER+/HER2−/High Proliferative, HER2+ and ER−/HER2−) by quantitative measurement of three genes (ESR1, ERBB2 and AURKA). Similar to the PAM50 subtype predictions, the molecular entities identified by the SCMGENE predictor were found significantly associated with survival outcome [6]. However, a direct head-to-head comparison between both predictors was not performed despite that fact that the concordance (i.e., κ score) between these two predictors was 0.59 (0.58–0.61), which is considered moderate agreement and similar to the κ scores obtained when histological grade is evaluated by two independent observers [7].

In this study, we compared the SCMGENE assignments and the research-based PAM50 assignments in terms of their ability to (1) predict patient outcome, (2) predict pathological complete response (pCR) after anthracycline/taxane-based chemotherapy, and (3) capture the main biological diversity displayed by all genes from a microarray.

## Materials and methods

### Clinical and gene expression data

We used the clinical (Supplemental file: jnci-JNCI-11-0924-s02.csv) and gene expression data (http://www.compbio.dfci.harvard.edu/pubs/sbtpaper/data.zip) as provided by Haibe-Kains et al. [6]. For survival predictions, we used distant metastasis-free survival as the endpoint since it provides the largest number of patients that can be evaluated across 13 datasets (CAL [8], EMC2 [9], DFHCC [10], MAINZ [11], MDA5 [12], MSK [13], NKI [14], TAM [15], TRANSBIG [16], UCSF [17], UNT [18], VDX [19] and VDX3 [20]). None of the datasets (or samples) used for survival (or response prediction) were used to derive the SCMGENE or the PAM50 subtype predictor.

To compare chemotherapy response data, we used the clinical data of one of the datasets (MAQC2 [GSE20194] [21]) evaluated by Haibe-Kains et al. [6], which is composed of 230 pre-treatment samples with annotated response data (pCR vs. residual disease [RD]) after neoadjuvant anthracycline/taxane-based chemotherapy. Samples that received trastuzumab were excluded.

### Combined microarray dataset

Eighteen Affymetrix and Agilent-based datasets (CAL [8], DFHCC [10], DUKE [22], EORTC10994 [23], EXPO [24], KOO [25], MAINZ [11], MAQC2 [21], MDA4 [26], MSK [13], NKI [14], PNC [27], STK [28], TRANSBIG [16], UNC337 [29], UNT [18], UPP [30] and VDX [19]) as provided in Haibe-Kains et al. [6] and with an appropriate distribution of ER+ (50–90 %, as defined by IHC) versus ER− tumors were combined into a single gene expression matrix. Probes mapping to the same gene (Entrez ID as defined by the manufacturer) were averaged to generate independent expression estimates. In each cohort, genes were median centered and standardized to zero mean and unit variance.

### Statistical analyses

Distant metastasis-free survival univariate and multivariate analysis were calculated using a Cox proportional regression model. Likelihood ratio statistics of subtypes defined by the PAM50 or the SCMGENE predictors were also evaluated after accounting for clinical–pathological variables (age at diagnosis, nodal status, and tumor size) and type of systemic adjuvant treatment (chemotherapy, endocrine, and none). Models were first conditioned on one predictor and the clinical–pathological variables, and then the significance of the other was tested. Chemotherapy response (pCR vs. RD) predictions of each variable were evaluated using univariate and multivariate logistic regression analyses. Finally, *R* ^2^ values of each predictor (SCMGENE or PAM50) for each principal component (PC) were calculated using a simple linear regression model. All statistical computations were performed in R v.2.8.1 (http://www.cran.r-project.org).

## Results

### Outcome prediction

To compare the ability of the SCMGENE and PAM50 assays to predict patient outcome, we performed Cox proportional hazard regression analyses using the entire combined dataset as provided by Haibe-Kains et al. [6]. In the multivariate model (MVA), both predictors were found significantly associated with distant metastasis-free survival (Table 1) and the Luminals A and B segregation of the PAM50 assay was found significantly associated with outcome, whereas the ER+/HER2−/Low Proliferative and ER+/HER2−/High Proliferative segregation of the SCMGENE predictor was not. Conversely, distant metastasis-free survival differences of the ER−/HER2− versus the ER+/HER2−/Low Proliferative groups were found significant, whereas the Basal-like versus Luminal A segregation was not.

**Table 1:** Distant metastasis-free survival Cox proportional hazards models of primary breast cancer patients

Variables	Univariate analysis				Multivariate analysis			
	HR	Lower 95 %	Upper 95 %	*p* Value	HR	Lower 95 %	Upper 95 %	*p* Value
Age (cont. variable)	0.989	0.983	0.996	0.003	0.996	0.988	1.003	0.257
Node status	1.176	0.851	0.992	0.063	1.695	1.315	2.184	<0.001
Tumor size T2–T4 versus T0–T1	1.305	1.104	1.541	0.002	1.242	1.042	1.480	0.015
Treatment (yes vs. no)	0.973	0.845	1.121	0.707	0.547	0.428	0.700	<0.001
PAM50								
Luminal A	1.0	–	–	–	1.0	–	–	–
Luminal B	1.797	1.503	2.149	<0.001	2.041	1.578	2.641	<0.001
HER2-E	2.677	2.120	3.380	<0.001	1.648	1.073	2.530	0.023
Basal-like	2.144	1.737	2.647	<0.001	1.312	0.812	2.121	0.268
Normal-like	1.073	0.670	1.718	0.769	1.024	0.572	1.835	0.936
Three-gene signature								
ER+/HER2−/Low Prolif	1.0	–	–	–	1.0	–	–	–
ER+/HER2−/High Prolif	1.852	1.531	2.241	<0.001	1.153	0.882	1.508	0.297
HER2+	2.785	2.196	3.533	<0.001	1.588	1.053	2.395	0.028
ER−/HER2−	2.536	2.041	3.150	<0.001	1.762	1.095	2.835	0.020

*HER2-E* HER2-enriched, *Prolif* proliferation, *HR* hazard ratio

**Fig. 1:**
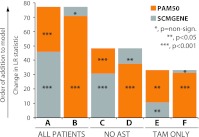
Distant metastasis-free survival likelihood ratio statistics of subtypes defined by the PAM50 or the SCMGENE predictors, after accounting for clinical–pathological variables (age at diagnosis, nodal status, treatment and tumor size). Models were first conditioned on one predictor and the clinical–pathological variables, and then the significance of the other was tested. (*A* **–** *B*) Entire combined dataset (*n* = 2,008), (*C*–*D*) subset of patients that did not receive adjuvant systemic therapy (*n* = 994), (*E*–*F*) subset of patients with HR+ tumors that received adjuvant tamoxifen-only (*n* = 491). Similar results are obtained if a term for dataset is included in the model

To compare the amount of independent prognostic information provided by each predictor, we estimated the likelihood ratio statistic of each predictor in a model that already included clinical–pathological variables (age, tumor size, treatment and nodal status) and the other predictor. The results revealed that the PAM50 subtypes provide a larger amount of independent prognostic information than the SCMGENE subtypes when using the entire cohort of heterogeneously treated patients (Fig. 1A, B). Similar results were observed when using the subset of patients that did not receive adjuvant systemic therapy (Fig. 1C, D), and in the subset of patients with HR+ tumors that received adjuvant tamoxifen-only (Fig. 1E, F).

### Chemotherapy response prediction

To compare the ability of the PAM50 and SCMGENE assays to predict response to chemotherapy, we evaluated the MAQC2 (GSE20194) [21] dataset included in Haibe-Kains et al. [6] analyses. This cohort is composed of 226 pre-treatment samples with annotated response data (pCR vs. RD) after neoadjuvant anthracycline/taxane-based chemotherapy (without trastuzumab for HER2+ disease). As shown in Table 2, although both assays predicted response in univariate analysis, the PAM50 assay was the only one to provide independent predictive information in the MVA model.

**Table 2:** pCR logistic regression models of the MAQC2 (GSE20194) [21] neoadjuvant breast cancer dataset

Variables	*N*	pCR rate (%)	Univariate analysis				Multivariate analysis			
			OR	Lower 95 %	Upper 95 %	*p* Value	OR	Lower 95 %	Upper 95 %	*p* Value
Age (cont. variable)	–	–	1.0	0.95	1.01	0.169	–	–	–	–
Tumor size										
T0–T1	23	35	1.0	–	–	–	1.0	–	–	–
T2–T4	203	19	2.3	0.92	5.86	0.076	0.4	0.13	1.23	0.111
PAM50										
Luminal A	66	3	1.0	–	–	–	1.0	–	–	–
Luminal B	66	9	3.2	0.62	16.47	0.164	5.2	0.68	37.97	0.108
HER2-E	28	46	23.5	5.25	105.36	<0.001	12.5	1.46	145.68	0.030
Basal-like	59	42	27.7	5.65	136.18	<0.001	25.3	2.64	255.95	0.005
Normal-like	7	0	0.0	0.00	–	0.988	0.0	0.00	–	0.988
Three-gene signature										
ER+/HER2−/Low Prolif	52	4	1.0	–	–	–	1.0	–	–	–
ER+/HER2−/High Prolif	85	8	2.2	0.45	11.23	0.325	0.6	0.08	4.62	0.633
HER2+	24	50	25.0	4.93	126.80	<0.001	3.9	0.34	46.46	0.275
ER−/HER2−	65	38	15.6	3.49	69.93	<0.001	0.9	0.09	9.97	0.954

*HER2-E* HER2-enriched, *Prolif* proliferation, *OR* odds ratio

Of note, the association of the PAM50 subtype with response was strengthened when PAM50 subtyping of the MAQC2 dataset was performed after median centering the PAM50 genes/rows (Supplemental Table 1). In fact, we and others have previously proposed median gene centering to minimize technical bias and allow the correct identification of the PAM50 intrinsic subtypes when appropriate representation of ER−, ER+, and HER2+ samples is available [31, 32]. Median gene centering of the UNC337 dataset before PAM50 or SCMGENE predictions also improved the survival classifications (Supplemental Fig. 1).

### Capturing the main biological diversity

**Fig. 2:**
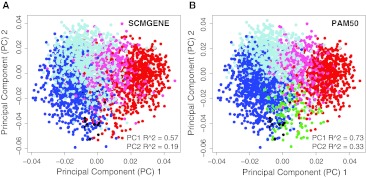
PC1 and PC2 loading plots of 3,316 samples using 18 Affymetrix and Agilent-based datasets taken from Haibe-Kains et al. [6]. Samples *colored* based on the **a** SCMGENE calls, or **b** PAM50 subtype calls. PC1 and PC2 *R* ^2^ values obtained from simple linear regression models are shown. Only datasets with >50 % and <90 % ER+ tumors were included in this analysis. *Blue* Luminal A or ER+/HER2−/Low Proliferative, *light blue* Luminal B or ER+/HER2−/High Proliferative, *pink* HER2-enriched or HER2+, *red* Basal-like or ER−/HER2−, *green* normal-like, *black* normal breast samples (only present in the UNC337 dataset [29]). For the UNC337 dataset, we colored samples based on the subtype calls obtained after median centering as shown in Supplemental Fig. 1

Finally, to compare both predictors in terms of their ability to capture the main biological diversity displayed by all genes in a breast cancer microarray, we first combined 18 datasets evaluated by Haibe-Kains et al. [6] and identified the two main *p*rincipal *c*omponents (PC1 and PC2). Compared to the SCMGENE subtypes, the PAM50 subtypes explained substantially more variation in gene expression for both PC1 and PC2 (Fig. 2a, b), with these components being especially prominent for the separation of the Luminal A (or ER+/HER2−/Low Proliferative) and Luminal B (or ER+/HER2−/High Proliferative) subtypes. To confirm these findings, we also evaluated all PCs in each normalized dataset provided by Haibe-Kains et al. [6] and observed that among 483 PCs significantly explained by either one of the predictors, the PAM50 explained 2.27 times more independent variation in expression than the SCMGENE assay.

## Discussion

Our results presented here, using the same data provided by Haibe-Kains et al. [6], suggest that (1) the SCMGENE and the PAM50 predictors should not be considered the same in terms of outcome prediction; (2) both provide independent prognostic information; (3) the amount of prognostic information provided by the PAM50 predictor is greater than the information provided by the SCMGENE predictor; and (4) the PAM50 assay is the only independent predictor of neoadjuvant chemotherapy response.

A potential explanation of our findings is that the biological diversity of breast cancer is better captured using the quantitative measurement of the 50 PAM50 gene set compared to the 3 genes of the SCMGENE assay. This finding is further supported by our previous data during the PAM50 assay development, where the minimum number of genes required to identify the intrinsic molecular subtypes, as defined by subtype classifications based upon the ~1,900 intrinsic gene list with a 93 % accuracy, was the final selected 50 genes [1]. In fact, gene sets with less than 50 genes showed significantly worse accuracies, particularly for tumors of the Luminal B and HER2-enriched subtypes (Supplemental Fig. 2). Importantly, only 33.3 % (12/36) of all microarray datasets evaluated in Haibe-Kains et al. [6] had all the PAM50 genes available, whereas 100 % of the datasets had all three genes of the SCMGENE assay, thus highlighting another caveat of this study.

In total, these analyses show that a combination of ER, HER2, and a single proliferation biomarker (i.e., AURKA) is prognostic, but is suboptimal to capture the biological diversity of breast cancers, which has similar implications for the capture of this biological diversity using IHC-based methods. Although a head-to-head comparison of both assays in terms of their clinical utility might be warranted in the future, our results suggest that classification of the major and clinically relevant molecular subtypes is better achieved using larger gene sets that capture a greater proportion of the biological diversity of breast cancers.

## Electronic supplementary material

Below is the link to the electronic supplementary material.

**Supplemental Table 1**. Logistic regression models of response in the MAQC2 neoadjuvant breast cancer dataset (*n* = 226) using the PAM50 subtype calls obtained after median centering the dataset as recommended in Perou et al. [31] and Lusa et al. [32]. **Supplemental Fig. 1**. PAM50 and SCMGENE subtype call differences obtained in the UNC337 dataset (GSE18229) with and without a platform normalization step. **a** Distribution of the SCMGENE and PAM50 subtype calls before and after median gene value centering of the dataset. Relapse free survival *curves* of the subtypes identified using the SCMGENE and PAM50 predictors obtained **b** before and **c** after median gene centering. **Supplemental Fig. 2**. Cross-validation performance on the PAM50 training dataset of different gene subsets of the starting ~1,900 genes, using the selected nearest centroid classification model. Note that the Luminal B, and HER2-enriched subtypes, are the most sensitive to the lower numbers of genes being used in the model, and thus if less than the 50 genes are used, these two subtypes accuracy will be the most compromised. (PDF 999 kb)
