# Supplementary material for: PAM50 assay and the three-gene model for identifying the major and clinically relevant molecular subtypes of breast cancer
Source: Breast Cancer Res Treat. 2012 Jul 3;135(1):301–6. doi: 10.1007/s10549-012-2143-0 (PMC3413822; doi:10.1007/s10549-012-2143-0)

Supplemental Table 1

Signatures	N	pCR rate	Univariate Analysis OR	Univariate Analysis Lower 95%	Univariate Analysis Upper 95%	Univariate Analysis p-value	Multivariate Analysis OR	Multivariate Analysis Lower 95%	Multivariate Analysis Upper 95%	Multivariate Analysis p-value
Age (cont. variable)	-	-	1.0	0.95	1.01	0.169	-	-	-	-
Tumor size										
T0-T1	23	35%	1.0	-	-	-	1.0	-	-	-
T2-T4	203	19%	2.3	0.92	5.86	0.076	0.4	0.12	1.78	0.093
PAM50										
Luminal A	75	1%	1.0	-	-	-	1.0	-	-	-
Luminal B	42	14%	12.3	1.43	106.32	0.022	55.7	2.11	1469.08	0.018
HER2-E	32	38%	44.4	7.29	431.35	<0.001	27.2	1.32	584.27	0.006
Basal-like	58	43%	56.1	5.44	362.21	<0.001	75.7	3.57	1691.25	0.033
Normal-like	19	11%	8.7	0.75	101.67	0.084	6.1	0.53	79.54	0.142
3-Gene Signature										
ER+/HER2-/Low Prolif	52	4%	1.0	-	-	-	1.0	-	-	-
ER+/HER2-/High Prolif	85	8%	2.2	0.45	11.23	0.325	0.2	0.01	2.89	0.213
HER2+	24	50%	25.0	4.93	126.80	<0.001	2.7	0.18	42.90	0.565
ER-/HER2-	65	38%	15.6	3.49	69.93	<0.001	0.5	0.03	8.45	0.632

Supplemental Figure 1

A

UNC-calls

Haibe-Kains calls

	ER-/HER2-	ER+/HER2- High Prolif	ER+/HER2- Low Prolif	HER2+
ER-/HER2-	62	25	0	8
ER+/HER2- High Prolif	14	54	28	18
ER+/HER2- Low Prolif	2	39	9	10
HER2+	9	17	3	7

Kappa score = 0.193

	Basal-like	HER2-E	Luminal A	Luminal B	Normal-like
Basal-like	52	3	0	15	13
HER2-E	9	14	6	13	4
Luminal A	1	12	33	18	0
Luminal B	7	12	56	7	2
Normal-like	9	0	4	10	5

Kappa score = 0.182

B

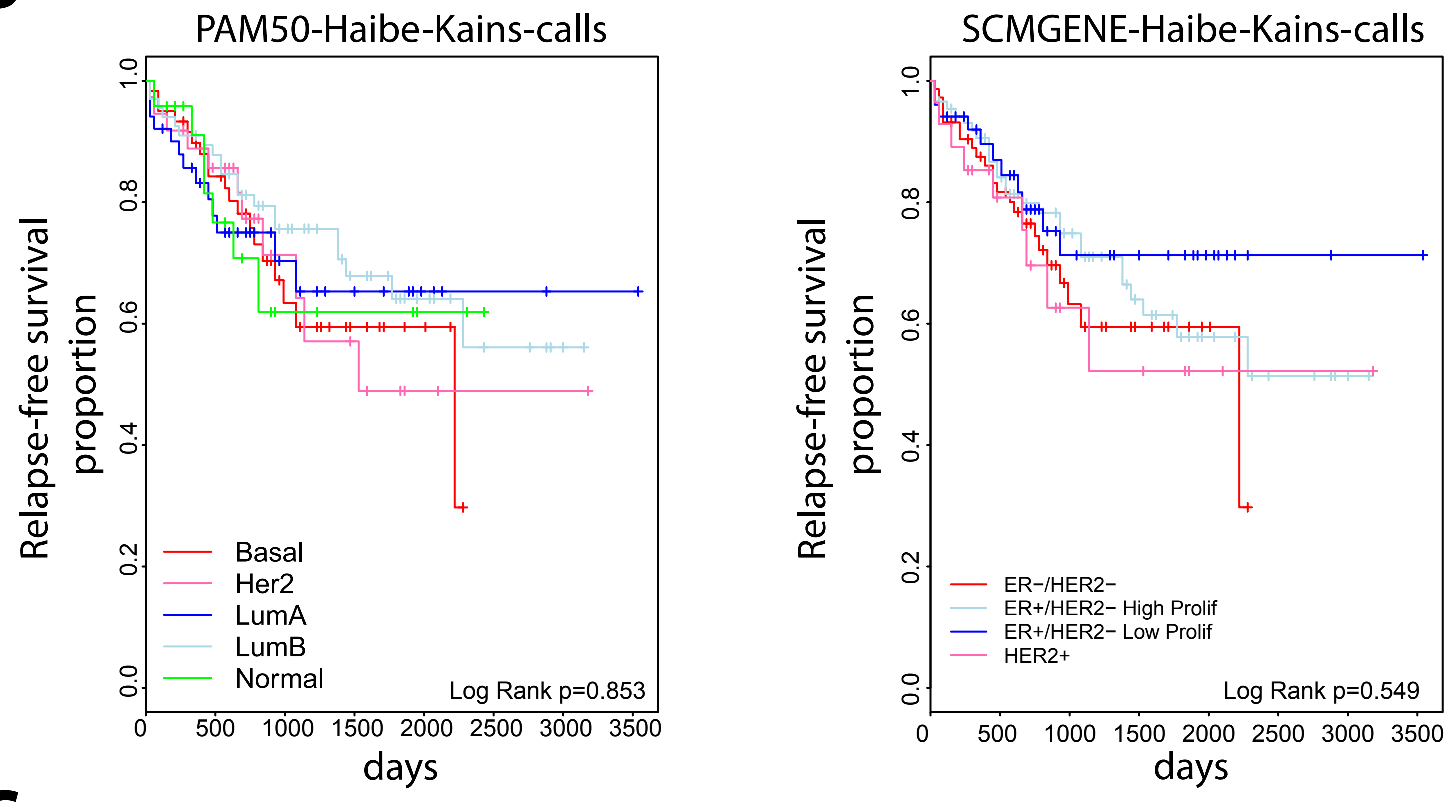

C

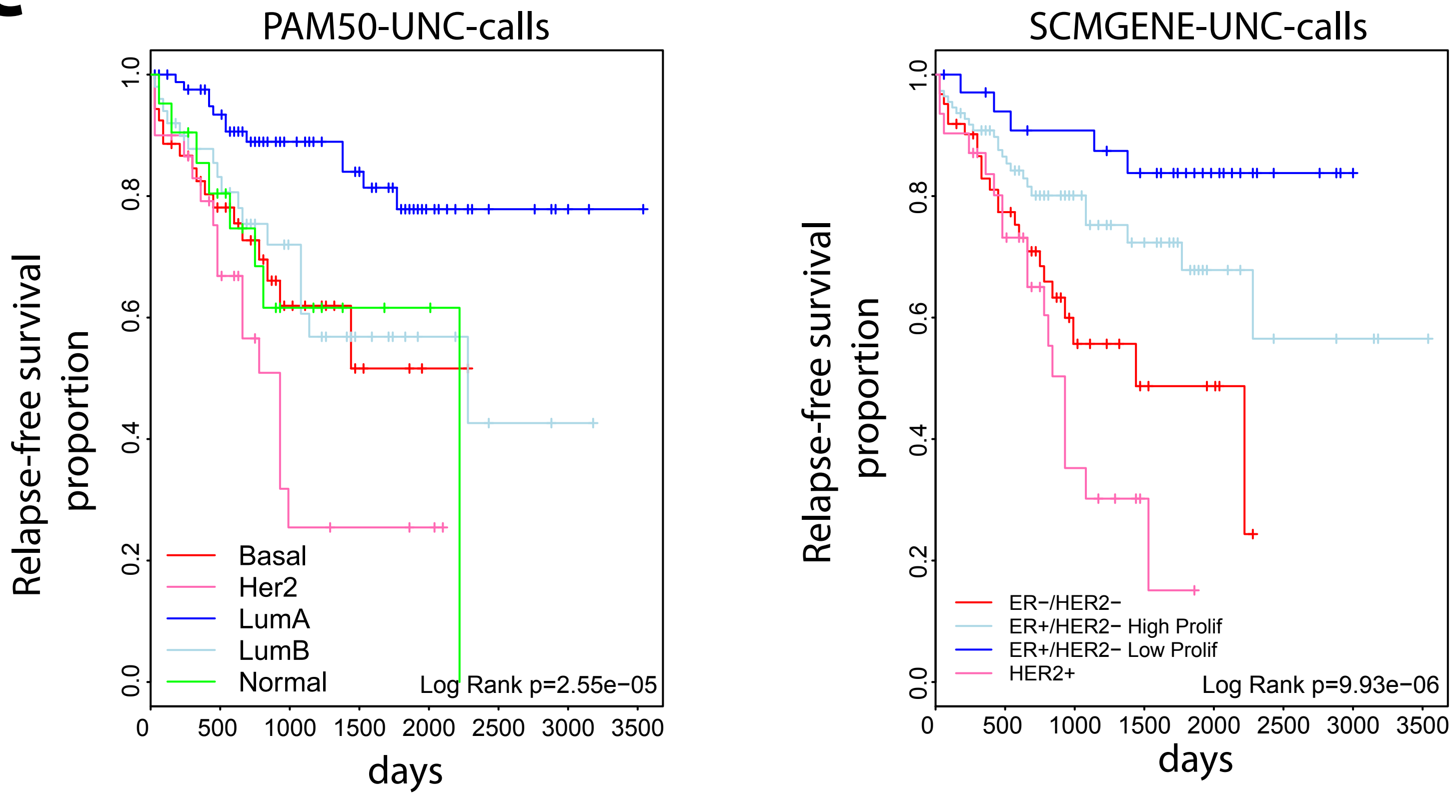

Supplemental Figure 2

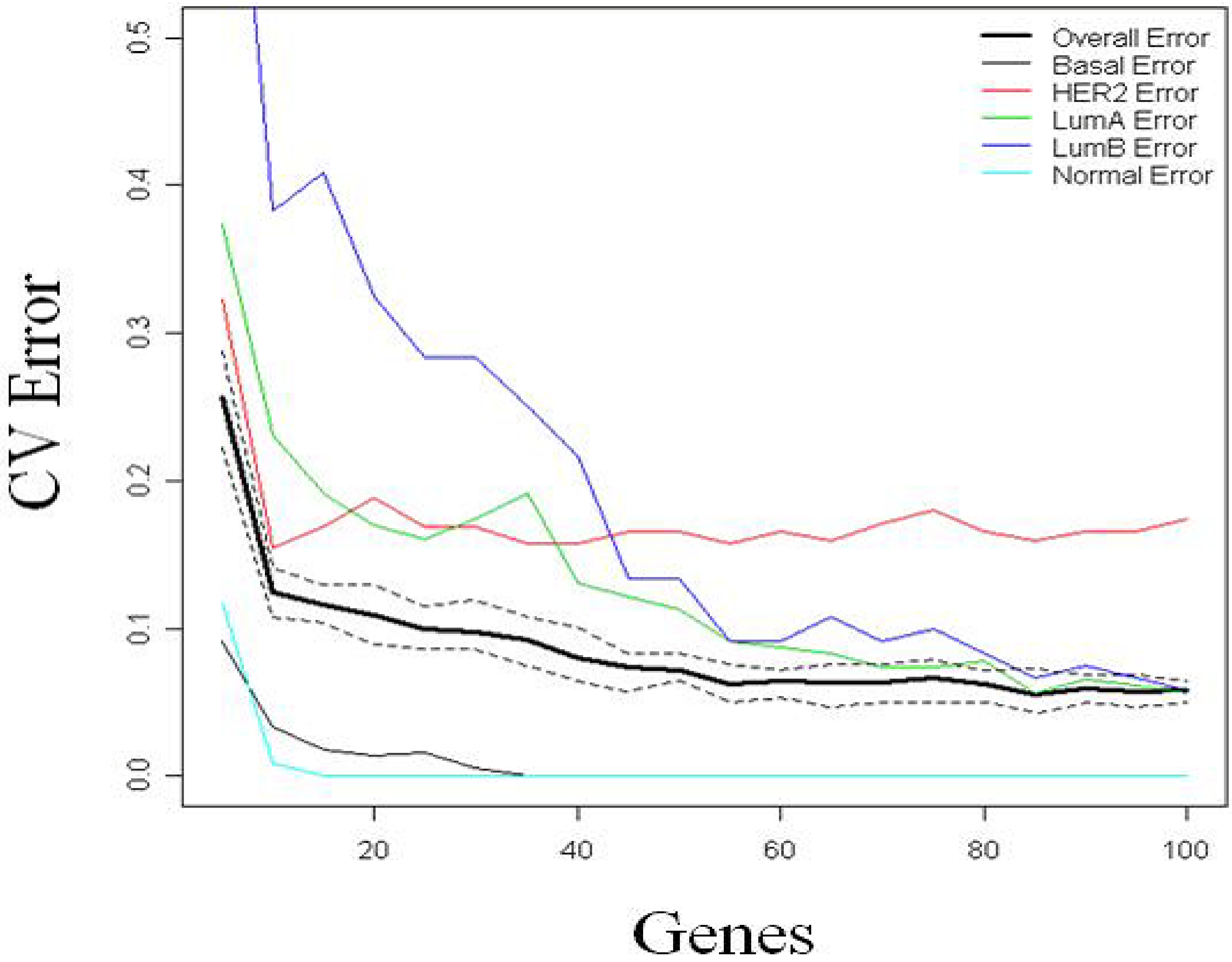

Supplement: Supplementary file 1 — Supplemental Table 1. Logistic regression models of response in the MAQC2 neoadjuvant breast cancer dataset (n = 226) using the PAM50 subtype calls obtained after median centering the dataset as recommended in Perou et al. [31] and Lusa et al. [32]. Supplemental Fig. 1. PAM50 and SCMGENE subtype call differences obtained in the UNC337 dataset (GSE18229) with and without a platform normalization step. a Distribution of the SCMGENE and PAM50 subtype calls before and after median gene value centering of the dataset. Relapse free survival curves of the subtypes identified using the SCMGENE and PAM50 predictors obtained b before and c after median gene centering. Supplemental Fig. 2. Cross-validation performance on the PAM50 training dataset of different gene subsets of the starting ~1,900 genes, using the selected nearest centroid classification model. Note that the Luminal B, and HER2-enriched subtypes, are the most sensitive to the lower numbers of genes being used in the model, and thus if less than the 50 genes are used, these two subtypes accuracy will be the most compromised. (PDF 999 kb) [file 10549_2012_2143_MOESM1_ESM.pdf]
